# Cloning, Expression, and Bioinformatics Analysis of the *AvFD1* Gene in *Amomum villosum* Lour

**DOI:** 10.3390/biology14050457

**Published:** 2025-04-24

**Authors:** Duo Wang, Yating Zhu, Shuang Li, Hongyou Zhao, Chongnan Wang, Qianxia Li, Yanfang Wang, Chunyong Yang, Ge Li, Yanqian Wang, Lixia Zhang

**Affiliations:** 1 Yunnan Key Laboratory of Southern Medicinal Utilization, Yunnan Branch of Institute of Medicinal Plant Development, Chinese Academy of Medical Sciences and Peking Union Medical College, Jinghong 666199, China; 2 Institute of Medicinal Plant Development, Chinese Academy of Medical Sciences and Peking Union Medical College, Beijing 100193, China; 3 Yunnan Key Laboratory of Sustainable Utilization of Southern Medicine, Yunnan University of Traditional Chinese Medicine, Kunming 650500, China

**Keywords:** *Amomum villosum* Lour., *flowering Locus D* gene, flowering regulatory gene

## Abstract

As a perennial medicinal plant in the *Zingiber* genus, *Amomum villosum* Lour. faces agricultural challenges due to its prolonged vegetative and reproductive growth phases, which hinder efficient pollination and delay fruiting. To address this limitation, the present study aimed to identify the *FD* gene involved in regulating flowering in *A. villosum* to provide a basis for research on the molecular mechanisms of early fruiting cultivars. Based on the differentially expressed gene *AvFD1* obtained from the transcriptome database of early fruiting plants and controls, specific primers were designed for PCR to clone the full-length sequence of *AvFD1*. The characteristics of the cloned *AvFD1* gene were analyzed using online bioinformatics software. The expression profiles of *AvFD1* in various tissues and in 1- and 2-year bearing *A. villosum* varieties were investigated by quantitative real-time PCR. This study successfully cloned the *FD1* gene sequence of *A. villosum*, marking the first reported characterization of this gene in the species. Tissue-specific expression analysis revealed significantly elevated *AvFD1* expression levels in stolon tips and flower buds compared to tender leaves, suggesting its potential role as a positive regulator of flowering initiation. The obtained sequence establishes essential molecular data for subsequent functional validation of *AvFD1* in *A. villosum*.

## Simple Summary

This study identified the *FLOWERING LOCUS D* (*FD*) gene in *Amomum villosum* Lour. to understand its role in flowering and early fruiting. The *AvFD1* gene was cloned by using PCR primers designed from transcriptome data of early-fruiting plants. Expression analysis showed the gene, while quantitative real-time PCR revealed that *AvFD1* expression was higher in stolon tips and flower buds compared to tender leaves, suggesting its relation to flowering pathways. As the first to sequence the *FD* gene in *A. villosum*, this work provides a foundation for functional validation and insights into early fruiting mechanisms in this species. These results provide genetic resources for molecular breeding of early fruiting cultivars, addressing the agricultural challenges caused by prolonged growth cycles.

## 1. Introduction

*Amomum villosum* Lour, a valued medicinal herb from the Zingiberaceae family, is a traditional Chinese medicinal plant called Sharen and is also known as condensed Misuosha. *A. villosum* has been extensively studied for its genetic diversity, evolution, and germplasm conservation [1,2]. It can regulate breathing, calm the fetus, warm the spleen, and prevent diarrhea. It grows in Guangdong, Yunnan, Guangxi, Guizhou, Sichuan, and other places in China. This herb, also known as *Amomi Fructus*, has been widely used in traditional Chinese medicine to address digestive issues such as diarrhea, bloating, and abdominal distention [3]. The therapeutic effects of *A. villosum* on inflammatory bowel diseases have been investigated with a focus on immune-related inflammatory cytokines and regulatory T cells as treatment targets [4]. The ethnopharmacological, phytochemical, and pharmacological properties of *A. villosum* have been thoroughly analyzed, highlighting its ability to warm the middle, dispel cold, regulate Qi, invigorate the spleen, and harmonize the stomach to prevent vomiting [5]. Additionally, the antioxidant aromatic compounds found in *A. villosum* have been identified, which signifies its potential medicinal and food value because of its diverse bioactive components [6]. Network medicine-based studies have elucidated the hepatoprotective effects of *A. villosum* [7]. Continuous cropping of *A. villosum* has also been studied, which revealed its effects on soil enzyme activities and physicochemical properties [8]. As a perennial herb, *A. villosum* has a long vegetative growth period before reproductive growth, which severely hinders its breeding research.

Flowering in plants is a complex process regulated by various pathways and environmental signals. The molecular events of floral initiation have been extensively studied in *Arabidopsis thaliana*, leading to a better understanding of the components required for signaling pathways, such as the brassinosteroid (BR) pathway [9]. Additionally, photoperiodic systems in plants respond to environmental and internal signals, with the CO family of transcriptional activators playing a crucial role in controlling flowering transition [10]. Temperature also regulates flowering time through the bHLH transcription factor, PHYTOCHROME INTERACTING FACTOR4 (PIF4), which induces flowering time genes [11]. The gibberellic acid (GA) pathway regulates plant height and flowering time, and OsNAC2 negatively regulates these traits by directly regulating key GA pathway genes in rice [12]. In *A. thaliana*, five distinct pathways have been identified to control flowering, including the photoperiod pathway, the vernalization/thermosensory pathway, autonomous floral initiation, the gibberellin pathway, and the age pathway [13]. Recent research has focused on the role of hormones, such as abscisic acid, auxin, cytokinin, salicylic acid, jasmonic acid, and ethylene, in regulating flowering; the findings revealed cross-regulation of these hormones with DELLA proteins, microRNAs, and transcription factors [14]. Moreover, studies on the CCT domain-containing gene family in rice have shown significant impacts of these genes on heading date, regional adaptation, and grain yield, further emphasizing the importance of genetic factors in flowering regulation [15]. The overexpression of the *SAMT* gene promotes early flowering in tobacco plants by regulating salicylic acid homeostasis, indicating the intricate role of hormone pathways in flowering regulation [16]. Overall, plant flowering pathways are complex and involve a network of interacting genetic and environmental factors that control the timing of plant reproduction. Further research on the molecular mechanisms and hormone regulation of flowering can provide valuable insights into plant development and crop productivity.

The transition from vegetative to generative growth in plants involves early changes in gene expression, as observed in the long-day plant *Sinapis alba* [17]. This transition is crucial for flower development, as reported by Goymer [18], which highlights the importance of timing and location for flower development in plants. The role of specific genes in regulating flowering time has been extensively investigated. For instance, Gorham et al. [19] identified a new role of the *FD* gene in maintaining reproductive meristem identity in an age-dependent manner in *A. thaliana*. Similarly, Muñoz-Fambuena et al. [20] found that the genetic inhibition of flowering differs between juvenile and adult citrus trees, which suggests the involvement of different molecular mechanisms. Environmental factors such as light intensity can also influence flowering time. Plantenga et al. [21] demonstrated that high light intensity accelerates potato flowering independent of specific flowering signals. Additionally, genetic mapping studies, for example, the study on capsicum species by Zhu et al. [22], have identified quantitative trait loci associated with flowering time and flower number per node. Manipulating specific genes related to flowering time can considerably affect plant development. Sriboon et al. [23] showed that knockout of *TERMINAL FLOWER 1* genes altered flowering time and plant architecture in *Brassica napus*. Pandolfini et al. [24] showed that the optimization of transgene action at the post-transcriptional level can facilitate the development of high-quality parthenocarpic fruits.

The FD protein belongs to the bZIP transcription factor family. In *A. thaliana*, the *FD* gene is mainly expressed in the shoot apical meristem (SAM) and is not affected by the circadian rhythm, photoperiod, or the activity of the *CO* gene. Under both long-day and short-day conditions, the FD mRNA level gradually increases as the plant grows and develops. The *FD* gene positively regulates the flowering time of plants. After the loss of *FD* gene function, the fd mutant *A. thaliana* exhibits a late-flowering phenotype, while *FD* gene overexpression promotes plant flowering [25]. In the plant flowering regulation process, the FT protein is a key regulator of the transmission of florigen signals from the leaves to the apical meristem. Following its expression in the leaf vascular tissue, the FT protein is transported to the apical meristem through the symplast pathway. In the apical meristem, the FT protein recognizes and binds to the FD protein, which is expressed only there, to form the FT-FD complex (florigen activation complex, FAC). This complex further activates the expression of the plant inflorescence meristem gene *SOC1* and the downstream flower meristem genes AP1 and LFY, thus promoting the transition of the plant from vegetative growth to reproductive growth and initiating the flowering process. The main function of CETS (CENTRORADIALIS/TERMINAL FLOWER1/SELF-PRUNING) proteins is to inhibit the flowering transition of plants and promote the indeterminate growth of meristems. In the apical meristem of plants, CETS proteins interact with FD proteins to form an “anti-florigen complex”. This complex can prevent or weaken the formation and function of the FT-FD complex, thereby inhibiting the expression of downstream flowering-related genes. Consequently, the plant remains in a vegetative growth state or experiences a delay in flowering time. Thus, the growth and development process of the plant is regulated, ultimately influencing the flowering time and architecture of the plant. The FD protein can enrich the functional diversity of the complex by selecting different interacting proteins and regulating different downstream target genes [26]. In conclusion, while substantial progress has been made in elucidating the role of the *FD* gene in flowering regulation, particularly through its interactions with *FT* and other regulatory elements, there remains a need for more extensive studies across diverse plant species. Future research should focus on the functional characterization of *FD* homologs in crops and their interactions with environmental factors to fully understand their contributions to flowering time and plant development. In summary, understanding the genetic and environmental factors that influence flowering time is essential for improving crop productivity and plant development. Thus, studies on gene expression, genetic mapping, and gene manipulation provide valuable insights into the complex regulatory networks that control flower development in plants. The current study is crucial for the breeding and genetic research of *A. villosum*. It not only provides a theoretical basis for the genetic improvement of *A. villosum*, but it could also serve as a reference for shortening the vegetative growth period and achieving early fruiting.

## 2. Materials and Methods

### 2.1. Materials, Reagents, and Main Instruments

*A. villosum* planted in the breeding base of Longmen Village, Mohan Town, Mengla County, Xishuangbanna Dai Autonomous Prefecture, Yunnan Province, China (21°17′36.75″ N, 101°32′59.75″ E) was used as the experimental material, and the flower buds and stolon tips of 3 plants with flower bud differentiation in the second year were used as the experimental material. In the control group, the stolon tips of 3 plants without flower bud differentiation in the second year were used for transcriptome sequencing. The top leaves of 3 seedlings with the same growth were taken from each group, and all the samples were frozen in liquid nitrogen and stored at −80 °C in a refrigerator.

The following reagents and instruments were used in the experiment: pMD™19-T plasmid (TaKaRa Bio, Kusatsu, Shiga, Japan), *Escherichia coli* DH5α receptor cells (Tiangen Biotech, Beijing, China), a Polysaccharide Polyphenol Plant Total RNA Extraction Kit (Tiangen), a Reverse Transcription Kit (Vazyme Biotech, Nanjing, China), TAK Link Conversion Kit (Toyobo Co., Ltd., Osaka, Japan), Taq Plus DNA polymerase (Tiangen), PrimeSTAR high fidelity enzyme (TaKaRa), a pipette gun (Eppendorf AG, Hamburg, Germany), a GL-20C-II cryogenic centrifuge (Flying Pigeon brand, Shanghai, China), DYY-10C electrophoresis apparatus, a nucleic acid electrophoresis system (Bio-Rad Laboratories, Hercules, CA, USA), a BioSpectrum 515 gel imaging system (UVP LLC, Uplands, MD, USA), a high-speed centrifuge (Thermo Fisher Scientific, Waltham, MA, USA), a T100 PCR instrument (Bio-Rad), a YKKY ice maker, a NanoDrop™ One UV-Vis spectrophotometer (Thermo Fisher Scientific), and a CFX96 Fluorescence Quantitative PCR Instrument (Bio-Rad).

### 2.2. Extraction and Reverse Transcription of Total RNA from A. villosum

Total RNA was extracted from *A. villosum* by using the Polysaccharide Polyphenol Plant Total RNA Extraction Kit (Tiangen), and the RNA concentration and absorbance value (A) ratios (A_260_/A_280_) at 260 and 280 nm were detected and recorded by an ultra-micro spectrophotometer. The total RNA of *A. villosum* was reverse transcribed by the reverse transcription kit to obtain cDNA. cDNA was detected by electrophoresis on a 1.5% agarose gel and stored at −20 °C.

### 2.3. Gene Cloning

Based on the preliminary transcriptome analysis by our research group, the CDS of the *AvFD1* gene (Wv08G0267) was used as the template. Primers FD1-F and FD1-R were designed using Primer5 software and sent to BGI Co., Ltd. (Guangzhou, China). for synthesis. After PCR cloning and tail addition, the target fragment was recovered and linked to the pMD 19-T carrier and transfected into *E. coli* receptor cells for culture. The plaque was selected for bacterial liquid PCR and electrophoresis detection, and the target-positive bacterial liquid was finally sequenced. Primer synthesis and sequencing were completed by BGI Co., Ltd. Table 1 provides details of the primer sequences.

### 2.4. Bioinformatics Analysis of Genes

Sequence comparison and search were conducted using the NCBI website (www.ncbi.nlm.nih.gov, accessed on 23 August 2024). The conserved domain analysis was used to analyze the CDS of the *AvFD1* gene. DNAMAN software (https://dnaman.software.informer.com/8.0/ accessed on 23 August 2024) was utilized for comparing nucleotide sequences. The relative molecular mass, isoelectric point, and average hydrophobicity of proteins were analyzed using the ProtScale online website (https://www.expasy.org/resources/protscale, accessed on 23 August 2024). The transmembrane domain of proteins was predicted online by TMHMM 2.0. The SOPMA (https://services.healthtech.dtu.dk/services/TMHMM-2.0/, accessed on 23 August 2024) and SWISS-MODEL (https://swissmodel.expasy.org/, accessed on 23 August 2024) online analysis websites were used to predict the two-dimensional and three-dimensional structures of proteins. NCBI-BLASTp (https://blast.ncbi.nlm.nih.gov/Blast.cgi, accessed on 24 August 2024), as well as Clustal X (University of Kyoto, Kyoto, Japan) and MEGA11.0 (The Pennsylvania State University, University Park, PA, USA) software, were utilized for searching homologous protein sequences and analyzing molecular phylogeny, respectively.

### 2.5. Fluorescent Quantitative PCR

The Primer-BLAST tool was used to design fluorescent quantitative PCR primers for the full-length sequence of the *AvFD1* gene; the primer sequences were as follows: F: CGGCTCTCTCTGCCTTTTCT, R: CTTCCACAGCTCCTCCATCG. The primers were synthesized by BGICo., Ltd. (Guangzhou, China). By using actin as the reference gene and Step 4.1 cDNA as the template, the system was constructed and analyzed by fluorescence quantitative PCR. The reaction procedure was as follows: Step 1, 95 °C for 30 s; Step 2, 95 °C for 5 s, 60 °C for 30 s, 40 cycles; Step 3, 95 °C for 10 s, 65 °C for 5 s, 95 °C for 5 s. Cq values were obtained by fluorescence quantitative PCR, and the fluorescence quantitative data were analyzed by the 2^−ΔΔCt^ method. The Primer-BLAST tool was used to design fluorescent quantitative PCR primers for the full-length sequence of the *AvFD1* gene. The primers were synthesized by Beijing Liuhe Huada Gene Technology Co., Ltd. By using actin-5 as the reference gene and Article 2.2 cDNA as the template, the system was constructed and analyzed by fluorescence quantitative PCR. The reaction procedure was as follows: Step 1, 95 °C for 30 s; Step 2, 95 °C for 5 s, 60 °C for 30 s, 40 cycles; Step 3, 95 °C for 10 s, 65 °C for 5 s, 95 °C for 5 s. Cq values were obtained by fluorescence quantitative PCR, and the fluorescence quantitative data were analyzed by the 2^−ΔΔCq^ method. The primer sequences are shown in Table 2.

## 3. Results

### 3.1. Amplification and Sequencing Results of the AvFD1 Gene

**Figure 1:**
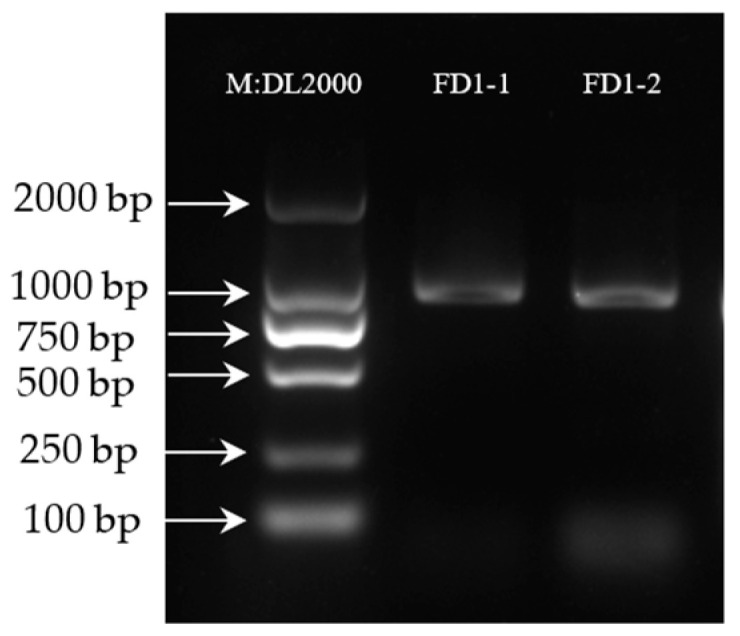
Agarose gel electrophoresis results of PCR-amplified products (Figure S1).

To determine the gene PCR results, the PCR products of the *AvFD1* gene were detected, and the results are shown in Figure 1. The DNA detection results confirmed that the positions of FD1-1 and FD1-2 were correct. The band was single, bright, and clear, and the amplified product length was consistent with the previous transcriptome sequencing results of 828 bp.

**Figure 2:**
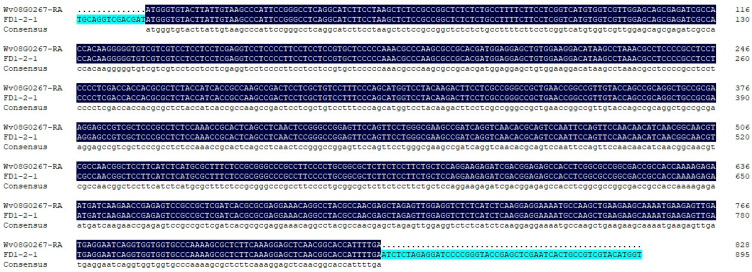
Sequencing results of the positive colonies PCR products.

Positive colonies for *Escherichia coli* were detected by PCR, and three positive colonies were sequenced by Beijing Liuhe Huada Gene Technology Co., Ltd. (Guangzhou, China). As shown in Figure 2, the sequencing results of the bacterial PCR products were consistent with the previous transcriptome sequencing results.

### 3.2. Bioinformatics Analysis

#### 3.2.1. Conservative Domain Analysis

**Figure 3:**
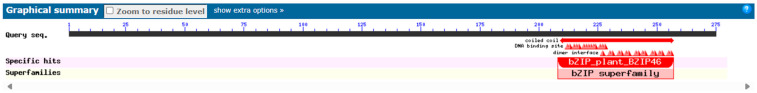
Prediction of the conserved domain of the AvFD1 protein.

Conservative domains are central to protein function and determine how proteins interact with other molecules. By identifying conservative domains, protein function can be inferred because certain domains are associated with specific biological functions. The prediction of the conserved domain of the AvFD1 protein based on the NCBI Conserved Domain Search database showed that all FD homologous genes from different species contain a conserved bZIP functional domain, indicating that *AvFD1* belonged to the bZIP transcription factor family (Figure 3).

#### 3.2.2. Analysis of the Physicochemical Properties of the AvFD1 Protein and Its Transmembrane Domain

**Figure 4:**
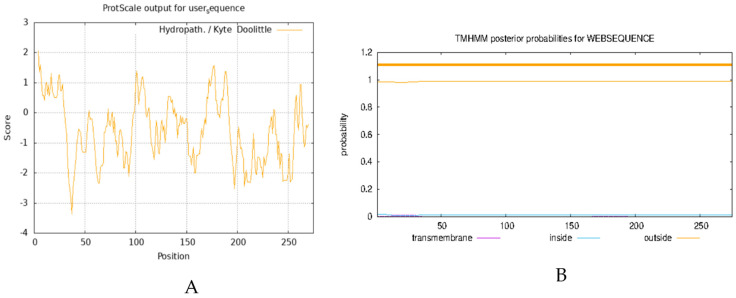
Prediction of hydrophilicity (**A**) and transmembrane domain (**B**) of the AvFD1 protein.

The analysis of the physicochemical properties of proteins enables the elucidation of the basic characteristics of proteins, such as molecular weight, isoelectric point, and amino acid composition, and provides the essential data for experimental design and protein function research. Transmembrane domain analysis reveals the functional properties of proteins in the cell membrane, such as signal transduction and material transport, which are essential for understanding cellular functions and physiological properties. Here, we analyzed the physicochemical properties of the AvFD1 protein and its transmembrane domain. The relative molecular mass, isoelectric point, and instability coefficient of the AvFD1 protein were 275, 9.80, and 75.49 > 40, respectively, thus suggesting that this protein was unstable. The hydrophilicity and hydrophobicity of the AvFD1 protein were predicted using the ProtScale tool of ExPASy (Figure 4A). The total average hydrophilicity was −0.561, indicating that the AvFD1 protein was hydrophilic. The AvFD1 protein showed the lowest peak at the 37th amino acid residue, with the lowest peak value of −3.356, and the highest peak at the 4th amino acid residue, with the highest peak value of 2.067. The lowest and highest peaks suggested that the structural stability of the AvFD1 protein at the 37th and 4th amino acid residues might be relatively low and high, respectively. We also predicted that the AvFD1 protein lacked a transmembrane domain (Figure 4B). These results demonstrated that the AvFD1 protein was an unstable hydrophilic protein with a moderate relative molecular mass and a slightly alkaline isoelectric point, together with specific differences in the structural stability of amino acid residues at different sites of the protein and absence of a transmembrane domain.

#### 3.2.3. Two-Dimensional and Three-Dimensional Structure Prediction of the AvFD1 Protein

**Figure 5:**
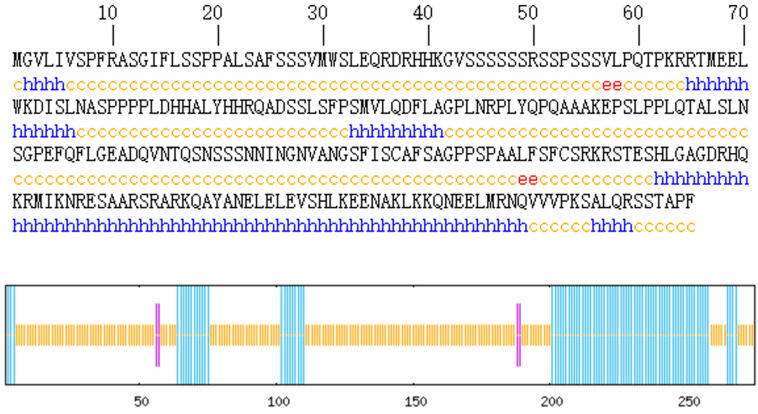
Secondary structure of the AvFD1 protein. The blue line segment and letters represent an α-helix; the purple line segment and red letters denote an extended chain; and the yellow line segment and letters signify a random coil.

Protein secondary structure prediction reveals the relationship between the amino acid sequence of the protein and its spatial structure. The secondary structure of the AvFD1 protein was predicted (Figure 5), and 87 amino acid residues (31.64%) were found to be involved in α-helix formation. Four amino acid residues (1.45%) participated in extension chain formation, while 184 amino acid residues (66.91%) were involved in random coil formation. Thus, the main structure of AvFD1 is α-helix and random coil. The most stable spatial structure of a protein is α-helix. Because the proportion of α-helix in the AvFD1 protein is only 31.64%, it is thought that the protein has poor stability.

**Figure 6:**
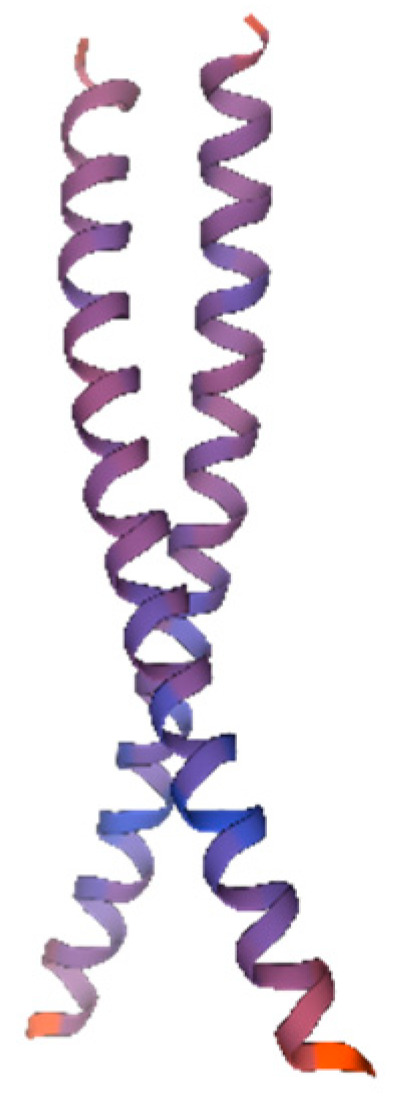
The 3D model of the AvFD1 protein. The blue and red parts of the figure represent the random coil and α-helix, respectively. The figure shows a homologous dimer. Proteins containing the bZIP domain are usually dimerized by leucine zippers and can form homologous or heterologous dimers [27].

Protein tertiary structure prediction provides a basis for further structural and functional research as well as molecular design. Therefore, we predicted the tertiary structure of the AvFD1 protein (Figure 6). The predicted AvFD1 protein had a motif of 1dh3.1.C (Transcription factor CREB [27]), an Oligo-State of Homo-dimer, a GMQE (global model quality estimation) value of 0.11, and a sequence similarity of 34.00%. The tertiary structure of the AvFD1 protein contains 51 amino acids, accounting for 18.21% of the total number of encoded amino acids, with low confidence.

#### 3.2.4. Phylogenetic Analysis of the AvFD1 Protein

**Figure 7:**
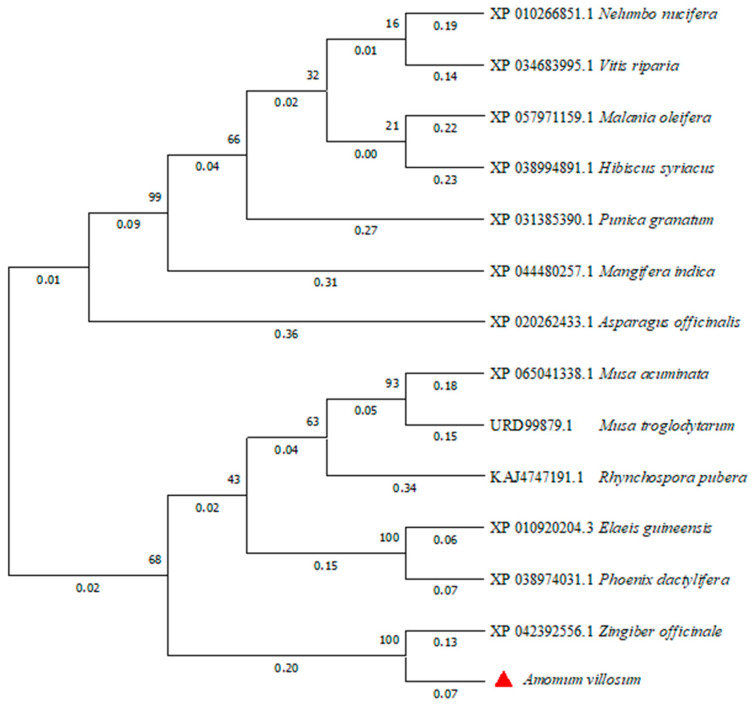
Phylogenetic analysis of the AvFD1 protein and FD1 proteins of other species.

The phylogenetic tree of FD proteins in different species was constructed by the neighbor-joining method using MEGA 11 software (Figure 7). The AvFD1 protein of *A. villosum* and the FD protein of *Zingiber officinale*, a member of the Zingiberaceae family, were located close to each other, thus indicating that they were closely related. This finding was consistent with evolutionary relationships.

### 3.3. Analysis of AvFD1 Gene Expression in Different Tissues of A. villosum

**Figure 8:**
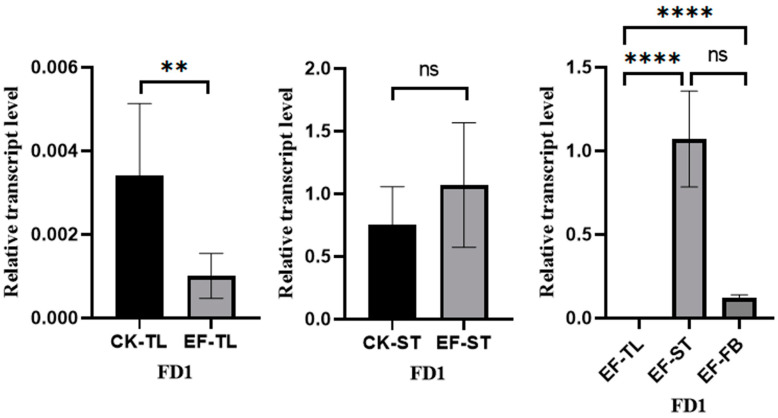
The relative expression levels of *AvFD1* in different strains and tissues. CK, control group; EF, early flower group; TL, tender leaves; ST, stolon tips; FB, flower buds. The error bars of the legend are the SEM (Standard Error of the Mean), and the sample size is 3. ns: Not significant (*p* > 0.05). **: Indicates statistical significance at *p* ≤ 0.01. ****: Indicates statistical significance at *p* ≤ 0.001.

The flowers and fruits of *A. villosum* grow on stolons. We conducted fluorescence quantitative PCR to detect the expression of *AvFD1* in different tissues of early fruiting individual plants (tender leaves, stolon tips, and flower buds) and non-early fruiting individual plants (tender leaves and stolon tips) (Figure 8). In the early fruiting *A. villosum* plants, *AvFD1* exhibited significantly higher expression levels in flower buds and stolon tips than in tender leaves. Moreover, the expression levels of *AvFD1* in the tender leaves and stolon tips of early fruiting plants were significantly lower and significantly higher, respectively, than those in the control group. These results indicate a significant correlation between *AvFD1* expression level and the flowering process. These findings imply that *AvFD1* is correlated with the flowering of early fruiting plants of *A. villosum*.

## 4. Discussion

*A. villosum* L., one of the four popular southern medicinal herbs in China, promotes gas flow, relieves abdominal pain, improves digestion, and eliminates dampness. As a perennial medicinal plant belonging to the genus *Zingiber*, *A. villosum* has a longer reproductive and vegetative period, leading to difficulties in pollination. Therefore, the flowering period of *A. villosum* should be carefully regulated. Flowering time is an important trait of *A. villosum*. It plays a crucial role in enabling the peak flowering period of *A. villosum* to avoid the arid climate. In the present study, the full-length CDS of *AvFD1* was cloned for the first time, and the gene was studied preliminarily through bioinformatics and expression analyses, which laid a foundation for further research.

Conserved domain analysis indicated that the AvFD1 protein belongs to the typical members of the bZIP transcription factor family. Sequence alignment analysis and phylogenetic tree analysis revealed that the *AvFD1* gene is highly similar to and relatively conserved among the *FD* genes that regulate flowering in other plants. qRT-PCR analysis revealed that the expression level of the *AvFD1* gene in the flowering tissues of *A. villosu*m was significantly higher than that in other tissues. These findings indicate that *AvFD1* belongs to the typical plant flowering pathway regulatory gene *FD* and is related to the flowering of *A. villosu*m. In *Arabidopsis thaliana*, *FD* is known to interact with FT to promote flowering in response to photoperiod changes [28]. This interaction underscores the importance of *FD* in the florigen pathway, which is pivotal for the timing of flowering in various plant species. In plants, the FD protein can bind to FT to form a florigen activation complex (FAC) or bind to the CETS to form an anti-florigen complex, thereby promoting or inhibiting plant flowering, respectively [26]. Bioinformatics analysis revealed that the AvFD1 protein lacks a transmembrane domain and has poor stability. Combined with the results of expression analysis, we speculate that AvFD1 may bind to the FT protein to form an FAC, thereby promoting the flowering of *A. villosu*m. plants. In some crops, mutations or changes in the expression level of genes related to the “florigen-anti-florigen” system lead to alterations in the flowering period and growth period of the crops; these mutations or changes provide important genetic resources for crop breeding [24]. In the present study, the expression level of the *AvFD1* gene in the reproductive parts of the early fruiting *A. villosum* plants was considerably higher than that in the reproductive parts of the non-early fruiting plants. This finding implies that the *AvFD1* gene may perform the function of regulating the flowering period or growth period in *A. villosum*. The fruits of *A. villosum* are used as a medicinal component in traditional Chinese medicine. However, currently, all the cultivated varieties of *A. villosum* take 3 to 4 years to enter the reproductive stage. This significantly increases the operational cost of plant breeders, making them less interested in growing *A. villosum*. In the future, we can regulate the reproductive period of *A. villosu*m by overexpressing the *FT* and *FD* genes, thereby enabling it to bear fruits earlier. The main cultivation area of *A. villosum*. is Yunnan Province, China. However, climate drought often occurs during the flowering period of *A. villosum* in this area, resulting in reduced production. In the future, we can control the flowering time of *A. villosum* by regulating the expression of the *FD* and *CETS* genes to achieve the goal of drought resistance. Notably, the predicted functions of these candidate genes require further experimental validation, such as transgenic assays in model plants or gene-editing studies (e.g., knockout or overexpression) in *A. villosum*, to conclusively determine their regulatory roles in flowering time control. Such functional characterization is essential to confirm the bioinformatic predictions and establish a reliable molecular basis for future applications in crop improvement.

## 5. Conclusions

In the present study, the *FD1* gene sequence of *A. villosum* was cloned for the first time. The expression analysis indicates that the *AvFD1* gene relates to flowering pathways, which provides a basis for the accurate verification and utilization of the gene function in the future. This finding is beneficial for the breeding research of the early fruiting and drought-resistant varieties of *A. villosum* and has a high application value. The specific physiological function of *AvFD1* in *A. villosum* remains unknown. Subsequent studies should develop transgenic *A. thaliana* plants to confirm the specific function of *AvFD1* in regulating the flowering time of *A. villosum*.

The identification of *AvFD1* holds immediate promise for molecular breeding programs aimed at developing early fruiting and drought-resilient *A. villosum* cultivars, which could significantly enhance crop productivity and sustainability. However, the precise molecular mechanisms underpinning *AvFD1’*s activity—including its interaction partners, signaling pathways, and epigenetic regulation—remain to be elucidated. To bridge this gap, we propose a dual-pronged investigative framework:

1. Functional Validation: Heterologous expression of *AvFD1* in model systems (e.g., transgenic *Arabidopsis thaliana*) to confirm its conserved role in flowering time regulation and assess phenotypic outcomes under controlled environments.
2. Mechanistic Exploration: Genome-wide analyses (e.g., ChIP-seq, RNA-seq) in *A. villosum* to identify downstream targets of *AvFD1* and characterize its regulatory network in native tissues.

Furthermore, field trials evaluating *AvFD1*-overexpressing lines under abiotic stress conditions (e.g., drought, salinity) could unlock its potential for developing climate-resilient varieties. By integrating these functional and applied approaches, this work opens new avenues for both fundamental research in flowering physiology and translational efforts in tropical crop improvement.

## Figures and Tables

**Table 1:** Sequence and Tm (melting temperature) value of *AvFD1* gene cloning primers.

Name	Sequence (5′-3′)	Tm Value (°C)
FD1-F	ATGGGTGTACTTATTGTAAGCCCAT	60.7
FD1-R	TCAAAATGGTGCCGTTGAGCT	62.9

**Table 2:** Sequences and Tm values of *AvFD1* gene and actin-5 qPCR primers.

Name	Sequence (5ʹ-3ʹ)	Tm (°C)
AvFD1(qPCR)-F	ATGGGTGTACTTATTGTAAGCCCAT	60.7
AvFD1(qPCR)-R	TCAAAATGGTGCCGTTGAGCT	62.9
Actin5-F	CGCATTGACGACCTCCAGTG	55.9
Actin5-R	TCTTCACCGCATGTGACAATCC	54.8

## Data Availability

Data are contained within the article.

## Supplementary Materials

The following supporting information can be downloaded at: https://www.mdpi.com/article/10.3390/biology14050457/s1, Figure S1: full western blot.

## Abbreviations

The following abbreviations are used in this manuscript:

BR pathway	Brassinosteroid pathway
bZIP	Basic Leucine Zipper
CCT domain	CO, CO-like, and TOC1 Domain
cDNA	Complementary DNA
CDS	Coding Sequences
CETS protein	CENTRORADIALIS/TERMINAL FLOWER1/SELF-PRUNING protein
CO gene	CONSTANS gene
FAC	Florigen activation complex
FD gene	Flowering Locus D gene
FT gene	Flowering Locus T gene
GA	gibberellic acid
mRNA	Messenger RNA
PCR	Polymerase chain reaction
PIF4	PHYTOCHROME INTERACTING FACTOR4
qPCR	Quantitative Real-time Polymerase Chain Reaction
SAM	shoot apical meristem
SAMT gene	Salicylic Acid Methyltransferase gene
