# Supplementary figures and images for: Cloning, Expression, and Bioinformatics Analysis of the AvFD1 Gene in Amomum villosum Lour
Source: Biology (Basel). 2025 Apr 24;14(5):457. doi: 10.3390/biology14050457 (PMC12108749; doi:10.3390/biology14050457)

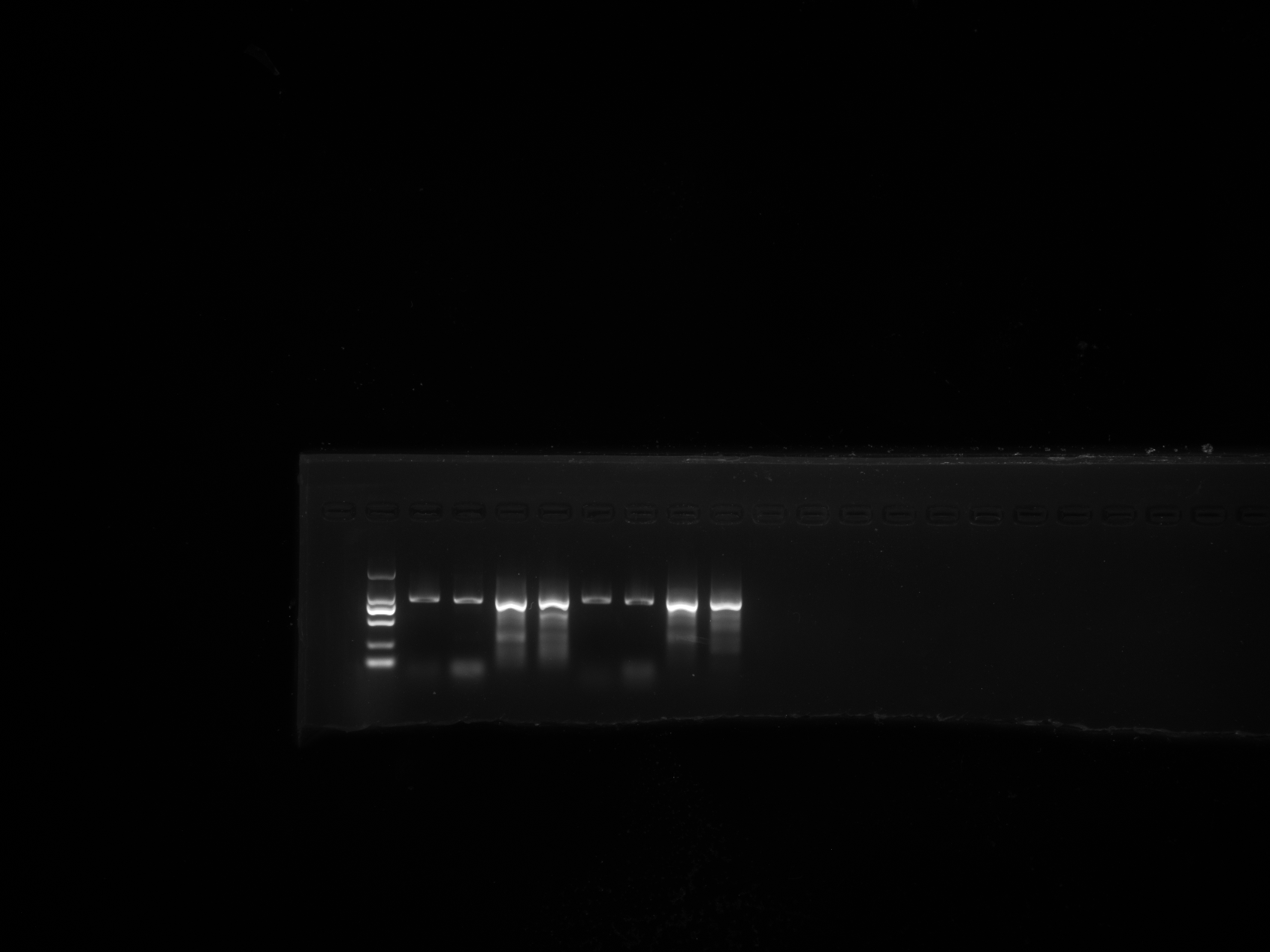

Supplement: Supplementary file 1 [file biology-14-00457-s001.zip › Figure S1-full western blot.TIF]
